# Sickle cell disease induces resistance to cutaneous carcinogenesis

**DOI:** 10.1186/s13023-020-1341-9

**Published:** 2020-03-06

**Authors:** Boutros Soutou, Patricia Senet, François Lionnet, Anoosha Habibi, Sélim Aractingi

**Affiliations:** 1 Faculté de médecine, Université Saint-Joseph, Beirut, 16-6830 Lebanon; 2 Department of Dermatology, Hôpital Tenon, Assistance Publique des Hôpitaux de Paris, Paris, France; 3 Department of Internal Medicine, Center for sickle cell disease, Hôpital Tenon, Assistance Publique des Hôpitaux de Paris, Paris, France; 4 Centre de référence des syndromes drépanocytaires majeurs, Unité des Maladies génétiques du globule rouge, Hôpital Henri Mondor, Assistance Publique des Hôpitaux de Paris, Créteil, France; 5 Faculté de médecine Paris 5 Descartes. Service de Dermatologie, Hôpital Cochin Tarnier, Paris. Equipe Cellules souches foetales, Inserm UMR S 938, Paris, France

**Keywords:** Sickle cell disease, Skin cancer, Skin ulcer, Hydroxyurea

## Abstract

**Background:**

While skin carcinomas are reported in chronic ulcers and in patients treated with hydroxyurea (HU) for myeloproliferative neoplasms, no skin carcinoma has been reported in patients with sickle cell disease (SCD), presenting chronic skin ulcers or treated with HU. The objective was to estimate the risk of cutaneous malignant transformation in SCD patients with prolonged leg ulcers or under HU therapy.

**Results:**

In this cross-sectional study, the cohort consisted of 1543 patients. In the first series, 29 patients presented a total of 53 ulcers lasting more than two years. The median age was 35 ± 8.4 years old. The median duration for a single ulcer was 9.2 ± 7 years. None of the examined ulcers showed any suspicious area of malignant transformation. In the second series, 187 patients treated with HU for more than two years were identified. The median age was 31.3 ± 9.9 years old. The median duration of treatment with HU was 6 ± 3.2 years. No skin carcinoma or actinic keratosis was recorded.

**Conclusions:**

This study showed that skin carcinogenesis did not occur in our series of SCD patients exposed to transforming events such as long term HU treatment or prolonged leg ulcers.

## Background

Sickle cell disease (SCD) is a genetic disorder of hemoglobin secondary to a mutation in the β-globin gene. It affects patients of African heritage classically living in Africa, Caribbean Islands or the Middle East. However, in the past decades, the situation has largely evolved. Indeed, due to the major therapeutic improvements, the survival of patients is actually reaching 58 years. In addition, the movements of populations have also changed the distribution of the disease. Therefore, SCD became the most frequent genetic disease in France and UK [1, 2]. In the U.S.A., the number of individuals with SCD may approach 100,000 [3].

The point mutation of SCD in the β-globin gene leads to the synthesis of defective hemoglobin that polymerizes when deoxygenated; consequently, erythrocytes become “sickled”, with decreased deformability. This results in vaso-occlusion and/or chronic intra vascular hemolysis, with a wide range of clinical complications affecting vital organs such as the lungs, the heart, the kidneys and the brain. In addition to supportive treatments, including red blood cells transfusion, the only drug that has shown efficiency is hydroxyurea (HU) [4]. Apart from its myelotoxic effect, HU indeed increases the foetal haemoglobin level, allowing, in many patients, a significant improvement of the disease severity. In such situations, patients can receive HU indefinitely, unless serious adverse effects occur.

As with other organs, the skin of patients with SCD may also be affected by tissue damage. Chronic leg ulcers (CLU) are the most frequent cutaneous complication of SCD. Geographical origin influences the occurrence of SCD leg ulcers, with a reported prevalence of 2.5% in the U.S.A. [5], 5.5% in France [6], between 1.5 and 13.5% in Africa [7], and above 40% in Jamaica [8]. Leg ulcers frequently present with severe pain, several complications such as infections, a high recurrence rate and a poor healing prognosis with a time-to-heal reaching several years [6–11].

Failing-to-heal leg ulcers of vascular or traumatic origin may transform into skin carcinoma after a prolonged course [12–14]. The mechanisms responsible for such neoplastic transformation remain unknown. Besides, in patients treated with HU for myeloproliferative neoplasms, skin carcinogenesis is frequently reported with actinic keratosis (AK) or squamous cell carcinomas (SCC) reaching up to 10% of recipients after 5 years treatment duration [15–17]. In our experience, as well as in the literature, there are no skin carcinomas- or even AK reported in patients with SCD whether presenting prolonged cutaneous ulcers or being treated with HU. Therefore, we hypothesized that skin cancer susceptibility might be very rare in SCD population. The aim of this study was to estimate the risk of cutaneous malignant transformation in patients with SCD presenting any of the classical carcinogenic events, namely CLU or HU treatment.

### Patients and methods

This is a cross-sectional study. The population consisted of all patients followed for SCD in two referral centres, Tenon and Henri Mondor Hospital, in Paris, France.

Concerning CLU cases, we selected all SCD-patients followed for non-healing leg ulcers, lasting for at least two years. We chose this stringent cut-off in order to sensitize the power of the study. Indeed, although carcinomas may arise as early as 6 months after ulcers, selecting a longer period should allow to depict more wound-associated cancers [18, 19]. Therefore, the files of all the SCD patients with leg ulcers were studied and only those eligible for the above criteria were selected. A letter was sent to these patients, explaining the aims of dermatological outpatient visit. Informed consent was obtained before study enrolment. One of two trained dermatologists (BS or PS) examined all the patients and collected data. These included age, sex, skin type, geographic origin, eventual immunosuppressing factors (drugs and/or diseases). A precise analysis of ulcers characteristics was performed: site, size, duration, number of relapses, and essentially any sign evoking a carcinomatous transformation. These consisted in irregular infiltrated margins, or growing granulation tissue. In these cases, a skin biopsy was performed to rule out a skin carcinoma.

Concerning the second cohort, we selected patients currently treated with HU for more than two years. Here also, we had to choose a minimal period even though in literature, the time to onset of AKs or skin carcinomas under HU is not well defined. Given the large number of patients in this population and the easiness to detect carcinoma or AK in these young patients, we decided to collect the data from detailed medical files in both centres. Age, sex, geographic origin, occupation, SCD type, immunosuppressing factors (drugs or diseases), HU treatment details (dose, duration), and of course the presence at any time in history of AKs, skin carcinoma or melanoma were recorded.

Data used for this study were stripped of personally identifiable information. The study was conducted in concordance with the French Ethical Rules as well as with the Declaration of Helsinki.

## Results

The cohort of SCD patients followed in the two referral centers at the time of the study consisted of 1543 patients, 1078 displaying the SS genotype from which we selected the SCD-prolonged ulcer group that corresponded to 29 patients presenting a total of 53 ulcers non healing ulcers for at least 2 years (prevalence: 2.6% of SS patients). Characteristics of these patients are shown in Table 1. These were 13 women and 16 men. The median age was 35 ± 8.4 years old (20–52). Twenty-two originated from Sub-Saharan Africa and 7 from the French West Indies. Most of them were of skin phototype VI. All ulcers were located as expected on the lower legs and the feet with a median size of 15.8 ± 23 cm^2^ (0.25–101.5). The median duration for a single ulcer was 9.2 ± 7 years (2–33) and the median number of recurrences for each ulcer was 5 ± 7.7 (0–30). Only one of these had occupational sun exposure. Five patients have received one or more skin grafts as part of ulcer treatment. Of note, 6 out of 29 patients with such CLU were also under HU treatment. Suspicious area of malignant transformation was never found in any case after precise clinical examination of the ulcer margins, the granulation tissue and the surrounding skin by the dermatologist. Consequently, no skin biopsy was performed. Follow-up of these cases never disclosed any sign of malignant transformation up to now.

**Table 1:** Sickle cell disease patients with non-healing leg ulcers, characteristics

Variable/Statistic	All patients (*n* = 29)
Sex (% female)	45
Age (years), median (range)	35 (20–52)
Skin type	
-VI(%)	90
-V(%)	10
Geographic origin	
-Sub-Saharan Africa (%)	76
-French West Indies (%)	24
SCD genotype, SS (%)	100
Ulcers (number)	53
-Site	
*leg (%)	15
*ankle (%)	72
*foot (%)	13
- Size (cm^2^), median (range)	15.80 (0.25–101.5)
- Duration (years), median (range)	9.2 (2–33)
- Number of recurrences, median (range)	5 (0–30)
-Malignant transformation	0

In the HU treated group, we identified 187 patients (117 women and 70 men) treated with HU for more than 2 years. Main characteristics are shown in Table 2. The median age was 31.3 ± 9.9 years old (11–77). Sixty-eight per cent originated from the Sub-Saharan Africa and 23.5% from the French West Indies. The large majority (93%) of these patients had homozygous SS genotype. The median duration of treatment with HU was 6 ± 3.2 years (2–17). The median daily dose was 1100 ± 300 mg (500–2000). There was no occupational exposure to sun (data not shown). Regarding immune-suppressive associated regimens, we found only 3 patients receiving low dose of prednisone and/or of methotrexate for rheumatic disease. Here too, for all these patients, no case of basal cell carcinoma, squamous cell carcinoma, melanoma, actinic keratosis (AK) or any other malignant tumour of the skin was recorded.

**Table 2:** Sickle cell disease patients treated with hydroxyurea, characteristics

Variable/Statistic	All patients (*n* = 187)
Sex (% female)	62.6
Age (years), median (range)	31 (11–77)
Geographic origin	
-Sub-Saharan Africa (%)	68.5
-French West Indies (%)	23.5
-Mediterranean region	6.4
-Madagascar	1.6
SCD genotype	
-SS (%)	93
-Sb (%)	3.7
-SC (%)	2.6
-Sdpunjab (%)	0.6
Hydroxyurea treatment	
- Duration (years), median (range)	6 (2–17)
- Daily dose (mg), median (range)	1100 (500–2000)
Skin cancer reported	0

## Discussion

This study shows for the first time that in individuals affected with SCD, skin carcinogenesis does not seem to develop despite exposure to various transforming events. Indeed, malignant transformation of CLU, as well as skin cancers related to HU therapy, were never found in our cohort of patients. In addition, to our knowledge, these complications have never been reported before in literature nor known by other colleagues in charge of these patients even in other continents. Finally, this study shows that 2.6% of SS patients had long lasting ulcers evolving for at least 2 years.

The results detailed above were provided through a robust study. Indeed, patients were included from a limited number of referral centres (two) for SCD where methods of patient management and medical records are similar. An exhaustive search of all medical files was done in order to retrieve data of patients to analyse. In addition, all leg ulcers were examined by trained dermatologists to assess the presence of any suspicion of transformation. Of note, our study did not perform systematic biopsies of ulcer tissue as this has already been done [10]. Such procedures are painful and may exacerbate ulcers in SCD affected patients. However, prolonged follow up did not show later any suspicious event. We can also note that, after the study ended, all the patients remained under regular medical observation until present time and no skin carcinoma was later reported; a fact that further enhances the findings of our study. A possible limitation of our study can be related to the impossibility of verifying the good compliance to HU prescription where a lower adhesion to treatment could interfere with the study outcome. However, all the examined ulcer patients as well as the HU patients had a regular specialist follow-up in both centres; the only fact that could warrant an acceptable compliance to treatment.

Malignant transformation of leg ulcers has already been evaluated. Although case reports are much more frequent than clinical series, studies have now been done [12, 18–21]. CLU of vascular origin can lead to the development of mainly well-differentiated SCC or verrucous carcinoma, usually after a long duration of evolution [12, 20–23]. Prevalence of malignant carcinoma reached 10% in a recent French prospective study of 144 patients presenting non-healing leg ulcers with a median duration of 6 years [12]. In a Swedish series, the relative risk for SCC in patients with CLU is 5.8 times as compared to controls [20]. In a British retrospective analysis, biopsy disclosed carcinoma in 4 of 17 ulcers with hyper-granulation and in 9 of 34 ulcers resistant to treatment [24]. Gil et al. showed that 10 out of 25 patients in a high suspicion group had malignancies [19]. Finally, it is important to note that such malignant transformation has also been described in African-American patients [25]. This is expected since mechanisms of wound-associated carcinogenesis do not rely on ultraviolet (UV) exposure.

If the risk of malignant transformation of non-healing SCD ulcers was similar to the lowest one reported in the literature, we should have seen at least 5 events of carcinoma in our cohort [12, 18–21, 23, 24]. Moreover, the duration of sickle cell ulcers was prolonged with a median of 9.2 years. Persisting inflammation with an elevated blood flow are shown to be similar in SCD leg ulcers and chronic venous ulcers [10]. Overall, the absence of any transformation despite prolonged ulceration seems to indicate an under-risk of skin carcinogenesis in response to wounding.

HU is potentially mutagenic and frequently carcinogenic for skin. Pre-carcinomatous lesions such as AK and subsequent SCC have been found to occur in patients treated with HU for myeloproliferative neoplasms [15]. Frequency of AK and cutaneous carcinomas was shown to reach 51 and 11% of treated cases respectively [16, 17]. Although median duration appears to be close to 46 months [26], other reports show that squamous cell carcinomas may develop as early as one to 6 months [27, 28]. Multiple lesions can also suddenly affect sun-exposed areas of light-coloured skin [29]. The SCD series that we report here shows again the absence of any of the classical occurring HU-related changes since there were neither AKs nor skin carcinoma. In accordance, reports of cutaneous adverse reactions in SCD patients treated with HU never noticed malignancies [30–32].

Mechanisms of skin carcinomas in sun-exposed areas are related to UV-induced molecular changes in humans as well as in mice models [33, 34]. HU induces cutaneous carcinogenesis nearly always in sun-exposed areas as well as this may occur in other drug-induced circumstances [35, 36]. The absence of any AK or SCC in our patients, in contrast with those treated for myeloproliferative neoplasms, may therefore be solely the consequence of SCD patients’ characteristics. These are younger, with a darker skin than patients with myeloproliferative neoplasms. Indeed, incidence of skin cancer is about 70 times lower in darkly pigmented persons than in light-skinned Caucasians, a direct result of inherent sun protection provided by increased epidermal melanin [37, 38]. However, HU may also induce carcinomas in other organs than the skin such as the oral cavity showing that oncogenic events may rise independently of UV exposure [39].

Although no accurate estimation of cancer in SCD is available in medical literature, one retrospective survey suggests that cancer incidence for patients with SCD might be roughly equivalent to that of the general African-American population [40]. Data on cancer development were indeed collected in 16,613 patients with SCD: cancer was diagnosed in 49 SCD patients with a median age of 34 years at malignancy diagnosis. Only 3 patients were under HU treatment. Nevertheless, since no cutaneous cancer was reported, it remains unclear if skin malignancies were listed in the survey form. Assuming that cutaneous carcinoma should have been reported if present, these results are in accordance with ours, suggesting a protection toward the risk of cutaneous carcinoma in SCD.

We believe therefore that patients with SCD do not develop skin cancer even when exposed to a well-known risk factor like CLU or HU trigger. Further clinical studies are required to better confirm this hypothesis. However, with the two previously cited potential protecting factors, namely young age and black skin, a comparative study is unrealizable in Europe. Relevant matching needs to be done on young black patients, without SCD, suffering from chronic ulcers or taking hydroxyurea, a condition nearly impossible. In addition, biological studies could help in understanding the mechanisms of such resistance. This could be eventually done using cultures of SCD keratinocytes with exposure to other than UV carcinogenic triggers and assessing the molecular response. However, this method would not take into account in vivo vascular or inflammatory changes. We recently developed a murine model for sickle cell ulcer [41]. This could be another tool to study the response to carcinogenic triggers in these mice.

## Conclusions

Our results indicate a peculiar resistance to cutaneous carcinogenesis in SCD patients. Depicting molecular pathways in skin response to carcinogenic triggers should bring original findings in skin cancer biology.

## Data Availability

All data generated or analysed during this study are included in this published article [and its supplementary information files].
